# Commencement of the Winter Sessions

**Published:** 1850-10

**Authors:** 


THE MEDICAL EXAMINER.

PHILADELPHIA, OCTOBER, 1850.

### COMMENCEMENT OF THE WINTER SESSIONS.

Before another number of our Journal shall be issued, our various Medical Schools will be actively engaged in the responsible duty of professional education. Already do we notice the appearance in our thoroughfares of those who have sought Philadelphia for her well known and justly appreciated advantages in medical instruction. Great as may be their anticipations of a rich harvest, we feel assured that they will not be disappointed, but that they will return to their homes at the close of the session more deeply than ever impressed with the conviction that here may be attained all that the most zealous and aspiring student can desire. It is predicted by those knowing in these matters that the classes at the various schools will be larger than at any previous season. We trust that their anticipations may be realized. It is fortunate that our extended territory will give the young aspirants room to flourish in after graduation.

